# T6SS: A Key to Pseudomonas’s Success in Biocontrol?

**DOI:** 10.3390/microorganisms11112718

**Published:** 2023-11-07

**Authors:** Edwin D. Navarro-Monserrat, Christopher G. Taylor

**Affiliations:** Department of Plant Pathology, Ohio Agricultural Research and Development Center, Wooster, OH 44691, USA; navarro.83@buckeyemail.osu.edu

**Keywords:** *Pseudomonas*, biocontrol, type VI secretion system

## Abstract

Bacteria from the genus *Pseudomonas* have been extensively studied for their capacity to act as biological control agents of disease and pests and for their ability to enhance and promote crop production in agricultural systems. While initial research primarily focused on the human pathogenic bacteria *Pseudomonas aeruginosa*, recent studies indicate the significance of type VI secretion (T6SS) in other *Pseudomonas* strains for biocontrol purposes. This system possibly plays a pivotal role in restricting the biological activity of target microorganisms and may also contribute to the bolstering of the survival capabilities of the bacteria within their applied environment. The type VI secretion system is a phage-like structure used to translocate effectors into both prokaryotic and eukaryotic target cells. T6SSs are involved in a myriad of interactions, some of which have direct implications in the success of *Pseudomonas* as biocontrol agents. The prevalence of T6SSs in the genomes of *Pseudomonas* species is notably greater than the estimated 25% occurrence rate found in Gram-negative bacteria. This observation implies that T6SS likely plays a pivotal role in the survival and fitness of *Pseudomonas*. This review provides a brief overview of T6SS, its role in *Pseudomonas* with biocontrol applications, and future avenues of research within this subject matter.

## 1. Introduction

Plant diseases are a major threat to our capacity to produce crops and consequently keep up with the food supply needed for our continuously growing human population. Among the many strategies applied in crop production for pathogen suppression, the use of biological control agents (BCAs) has garnered attention because it provides a means of reducing the use of chemical pesticides [1,2]. Over the past two decades, the agricultural protection industry has been shifting its research and development efforts toward biocontrol microorganisms and biopesticides derived from natural materials, such as plants, animals, bacteria, or biologically derived minerals [3,4]. Many plant-associated microorganisms act as natural antagonists against phytopathogens and hence have been extensively studied as BCAs. One such group of microorganisms is the bacterial members of the genus *Pseudomonas*.

Gram-negative bacteria belonging to the genus *Pseudomonas* are well known for their genetic, metabolic, and phenotypic diversity. These occupy various ecological niches and can exhibit pathogenic and commensal associations with their host. In the context of plant-associated *Pseudomonas*, several species have been shown to be beneficial to plant development and/or the biocontrol of phytopathogens. Such is the case of well-studied species such as *P. putida*, *P. protegens*, *P. fluorescens*, and *P. chlororaphis*, all of which can possess one or more traits that allow them to be successfully deployed as BCAs [5,6,7,8,9,10]. These traits include: (1) their ability to grow rapidly and be cultured in simple media, (2) their competence as colonizers of plant tissue (mainly root tissues), (3) their competitive fitness in various environments, and (4) their capacity to produce a wide range of bioactive metabolites that either directly or indirectly help promote plant development and/or inhibit the biology of phytopathogens.

Root colonization and production of metabolites with potential biocontrol purposes have been topics of focus in *Pseudomonas* biocontrol studies. Indeed, *Pseudomonas* have been shown as being capable of producing a wide range of metabolites with pathogen suppression potential. They produce chemicals with antibiotic properties such as pyrrolnitrin, phenazines, and 2,4-diacetylphloroglucinol (DAPG) [7,8,9,10,11]; lipopeptides such as masselotide A, and orfamide [12]; volatile compounds such as hydrogen cyanide (HCN) [13] and dimethyl disulfide (DMDS) [14]; and metabolites for niche competition such as siderophores, which are used for sequestering iron, limiting nutrient availability for other competing microbes [15].

In addition to small molecules, *Pseudomonas* are also well known for their ability to secrete larger, more complex molecules via multi-gene secretion systems. Bacterial secretion systems are molecularly complex protein machines that facilitate the transport of various molecules, such as DNA, proteins, and toxins to the environment or directly into another biological interactor and play a pivotal role in modulating host, environment, and intermicrobial interactions [16]. To date, nine secretion systems have been identified in bacteria, and six (type I, II, III, IV, V, and VI) have been reported in *Pseudomonas* [17,18]; see Figure S1. Of relevance to this review, the more recently described type VI secretion system (T6SS) is a harpoon-like apparatus that confers competitive advantages to many Gram-negative bacteria. In this review, we present an overview of T6SS and its known mechanism and functions and we highlight relevant studies involving T6SSs in non-pathogenic, biocontrol *Pseudomonas*. We also discuss the implications of these studies for the use of *Pseudomonas* as biocontrol agents and future avenues of research relating to T6SSs in biocontrol *Pseudomonas*. 

## 2. Overview of T6SS: Discovery, Structure, Mechanism of Action, Associated Functions, and Classification

### 2.1. Discovery

The first evidence of type VI secretion systems came in 2003 when Blandergroen et al. [19] reported that the mutation of a gene cluster, then termed *imp* (for impaired nodulation), in *Rhizobium legumisarum* led to a decrease in nodulation and nitrogen fixation. The mutation occurred in *impJ*, which is now known to be *tssK*, an important structural component of the T6SS. The first description of T6SS came in a set of studies by members of the John Mekalanos group [20,21]. Pukatzki et al. [20] reported that the cytotoxicity of *Vibrio cholerae* towards *Dictyostelium* amoebae was mediated by a set of genes then termed “VAS” genes (for virulence-associated secretion), which encoded a protein secretion machinery. The study also reported the secretion of the proteins hemolysin coregulated protein (Hcp) and valine-glycine repeat protein (VgrG), which were needed for contact-dependent cytotoxicity and are now known to harbor T6SS effectors. The second study by Mougous et al. [21] described T6SS in *Pseudomonas aeruginosa*, providing evidence of active Hcp (TssD) secretion in the lungs of patients with cystic fibrosis and involvement of ClpV (TssH) ATPase in the T6SS-mediated translocation of Hcp.

### 2.2. T6SS Structure and Mechanism of Action

**Figure 1:**
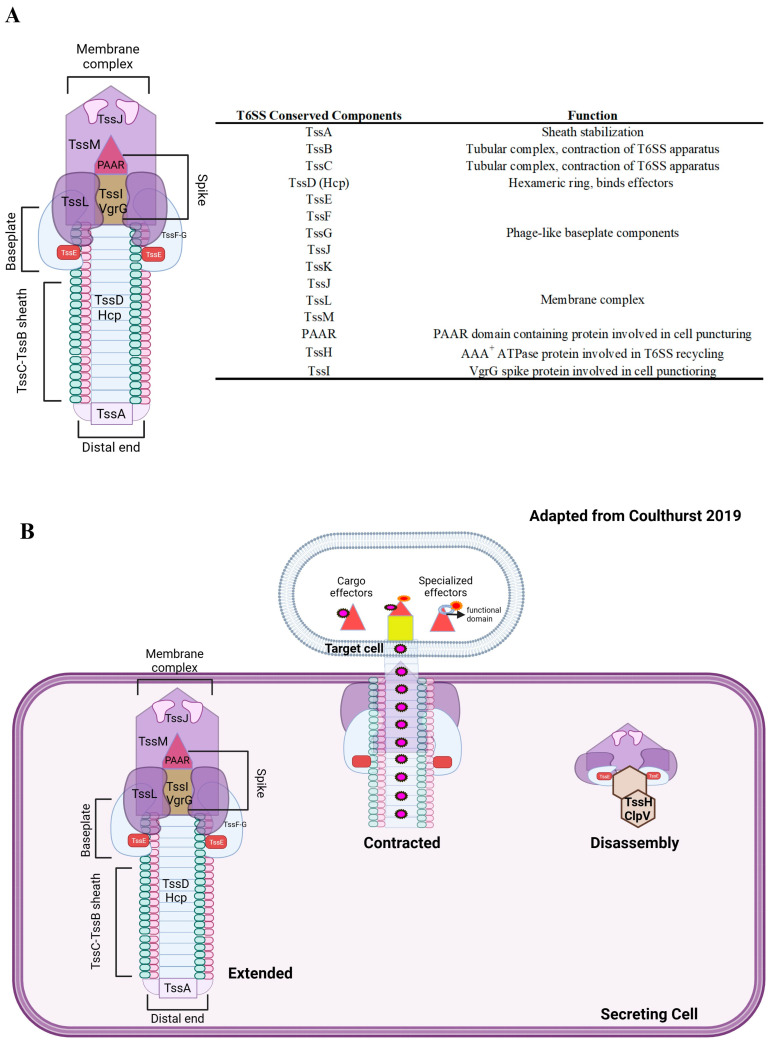
(**A**) T6SS structure, core components, and functions. (**B**) Schematic representation of T6SS effector delivery, adapted from Coulthurst [25]. A fully assembled T6SS contracts its TSSB/C sheath to facilitate the transfer of TssD-TssI-PAAR spike components out of the cell and into the target cell for effector delivery. T6SS effectors can be cargo or specialized effectors, as described in the main text. After contraction, TssH/ClpV-mediated disassembly of the T6SS prepares the system for subsequent firing events. Created with BioRender.com (accessed on 8 May 2023).

We now know T6SSs as contractile protein nanomachines utilized to translocate effectors into prokaryotic and eukaryotic cells [22,23]. T6SSs resemble bacteriophages in several ways and are often described as being phage-like (Figure 1A,B). Various components of the T6SS have structural homology to those found in bacteriophages. For example, both Hcp (TssD) and VgrG (TssI) proteins have been shown to have structural homology to the gp19 tube and gp27_3_-gp5_3_ needle-like complex found in T4 bacteriophage [23,24]. 

Structurally, the T6SS consists of 13–14 core components termed TssA-TssM for type VI secretion and a PAAR (for proline-alanine-alanine-arginine repeats) structural component that caps the VgrG/TssI spike (Figure 1A). Collectively, these components form three main complexes: the membrane complex (TssJML), the phage-like baseplate (TssKEFG) and spike (TssI, PAAR), and the contractile sheath (TssBC) [22,23,24,26,27,28,29]. Each component plays a specific role in T6SS assembly, firing, disassembly, and/or in the recycling of T6SS components. T6SSs are known for their contact-dependent nature, where the effector delivery process requires direct cell–cell contact. The mechanism for effector delivery (summarized in Figure 1B) involves contraction of the extended sheath in the assembled T6SS, which propels the Hcp-VgrG-PAAR structure into the target cell. After contraction, TssH ATPase-mediated disassembly of the T6SS occurs and prepares the system for another firing event. 

Newer research has also shown the contact-independent translocation of effectors into other target prey cells by binding to outer membrane components present in those target cells [30]. Additionally, it has also been shown that T6SSs can secrete effectors extra- and intra-cellularly for the contact-independent uptake of metal ions [31,32]. These studies broaden the scope of T6SS-associated functions and challenge how we traditionally define T6SS. Instead of defining such as a nanomolecular weapon with contact-dependent prerequisites for activity, they may now be defined as a more versatile system that is involved in a myriad of functions that can either be of a contact-dependent or contact-independent nature.

### 2.3. T6SS-Associated Functions

Initial descriptions of the T6SS in Pukatzki et al. [20] and Mougous et al. [21] suggested that it played a role in host virulence and eukaryotic interactions. Indeed, several studies have reported T6SS-related eukaryotic and anti-host interactions. Some examples include T6SS-assisted internalization of *P. aeruginosa* into non-phagocytic [33] and epithelial cells [34], which suggest a role in host invasion. Despite studies depicting the relevance of T6SS in eukaryotic interactions, the discovery and descriptions of a plethora of T6SS effectors (T6SEs) with antibacterial activity suggest that this system functions primarily as an antibacterial weapon [24]. Antibacterial T6Ses can cause growth stasis, disruptions in biofilm formation, and cell death [35,36] against competing bacteria. More recently, T6SSs have been implicated in contact-independent metal uptake, DNA uptake, and antifungal activity [37,38]. These continue to expand our knowledge on T6SS-associated functions and highlight the potential competitive advantages of T6SS-bearing bacteria. 

### 2.4. T6SS Effectors and Immunity Proteins

T6SS effectors can be divided into two categories: cargo effectors and specialized effectors. Specialized effectors have a functional domain and one of the TssI/TssD/PAAR domains, while cargo effectors interact non-covalently with TssI/TssD/PAAR domains [25,39]. Most of the T6SEs that have been identified to date are involved in interbacterial competition, with many of them functioning as inhibitors against other competing bacteria. These effectors are categorized based on their function and targets. Antibacterial T6SEs can target nucleic acids through nucleases, the cell wall through amidases and glycoside hydrolases, and the inner cell membrane through phospholipases and pore-forming proteins [39,40,41,42,43]. The diversity of effectors reflects T6SS’s versatility as a nano-molecular weapon.

T6SEs are often encoded within the vicinity of T6SS clusters or throughout the genome in association with orphan *hcp*, *vgrG*, or *PAAR* genes (often referred to as orphan islands). These effectors often co-occur with cognate immunity proteins that confer resistance. This allows for the recognition of kin cells and can also confer resistance to other T6SS-using bacteria.

### 2.5. Classification of T6SS Clusters

Phylogenetic analysis of T6SS loci in various Gram-negative bacteria has yielded valuable insights into understanding the evolutionary origins of the type VI secretion system. A set of studies by Bingle et al. [44] and Boyer et al. [45] gave initial insights into the evolution and phylogenetics of T6SS. Bingle et al. [44] performed a bioinformatic analysis of a widespread dataset of Proteobacteria and identified that T6SSs were present in more than 25% of the genomes. The study also used concatenated TssB/TssC sequences to generate phylogenetic trees and found that T6SS clusters in Proteobacteria could be divided into four groups (I-IV). Boyer et al. [45] identified 13 conserved clusters of orthologous groups (COGs) in T6SS and used these for subsequent phylogenetic analysis. The results showed that the Proteobacterial T6SS clusters can be divided into five groups (I–V). T6SSs with different evolutionary origins to those of Proteobacteria were also uncovered in *Bacteriodetes* [46], *Francisella* [47], and *Amoebophilus asiaticus* [48]. Hence, T6SSs are now classified as T6SSi for the canonical Protebacterial T6SS, T6SSii for those found in *Francisella*, T6SSiii for those found in *Bacteriodetes*, and T6SSiv for those found in *Amoebophilus asiaticus* [48,49].

## 3. Bacterial T6SS-Mediated Interactions with Plant Host

The role of T6SS in mediating bacteria–plant interactions remains largely unclear as, to date, no T6SS effector has been reported to directly interact with plant cells. Despite this, several studies report that rendering the T6SS non-functional can impair bacteria–plant interactions. Observations of impaired nodulation in *Rhizobium* produced by deletion of the gene encoding for the T6SS structural component, TssK [19], suggest that T6SS may potentially be involved in this process; however, more work would be needed to confirm this hypothesis. Additionally, the absence of T6SS activity has been shown to reduce the pathogenicity of *Ralstonia solanacearum* on tomato [50] and *Pantoea ananatis* on onion leaves [51]. Understanding the underpinning mechanisms by which T6SS can mediate symbiotic, pathogenic, and potentially beneficial bacteria–plant interactions is an interesting avenue of T6SS research that can yield valuable insights into the interplay between bacteria and their plant host.

## 4. The *Pseudomonas* T6SS

As members of the Gammaproteobacteria class, the T6SS found in *Pseudomonas* is the canonical Proteobacterial T6SS. *Pseudomonas* spp. have been instrumental in understanding the structure, phylogenetics, functioning, and regulation of the Proteobacterial T6SS. In particular, *P. aeruginosa* has served as a model organism for T6SS-related studies. In this section, we review the classification and evolution of *Pseudomonas* T6SS clusters, the use of *Pseudomonas aeruginosa* as a model organism for T6SS studies, and the prevalence of T6SS in *Pseudomonas*.

### 4.1. Phylogenetics of T6SS Loci in Pseudomonas

In *Pseudomonas*, a phylogenetic analysis of T6SS loci using eleven conserved T6SS COGs by Barret et al. [52] revealed that these can be classified into six distinct clusters. This largely fits the classification system by Boyer et al. [45], with the addition of cluster IV being subdivided into two groups. Furthermore, genomes of individual *Pseudomonas* often contain more than one T6SS locus and, in some cases, these fall into different evolutionary clusters (Figure S2), providing evidence that these were acquired through horizontal gene transfer. Many studies have subsequently used the proteobacterial T6SS classification nomenclature proposed by Boyer and Barret. The relationship between cluster classification and T6SS effector/immunity protein content remains unclear. However, it has been shown that *Pseudomonas* bearing multiple T6SS clusters can have differing T6SS activity among the different clusters [33,53].

### 4.2. Pseudomonas aeruginosa as a Model Organism for T6SS

*Pseudomonas aeruginosa* is an opportunistic human pathogen most commonly associated with patients who have cystic fibrosis. Since the initial discovery of T6SS in *P. aeruginosa*, it has continued to be used as a model bacterium for T6SS and has proven itself essential to the understanding of the structure, functioning, and regulation of this secretion system [54]. *P. aeruginosa* PA01, a model strain, encodes three T6SS clusters termed H1-T6SS, H2-T6SS, and H3-T6SS. These clusters have been shown to have evolutionarily distinct origins with different associated functions [41]. H1-T6SS has been mostly implicated in antibacterial activity, while H2-T6SS and H3-T6SS have been associated with nutrient acquisition and environmental adaptation [34,55,56].

The extensive literature on *P. aeruginosa* T6SS has been reviewed elsewhere [54,57]. However, we briefly introduce *P. aeruginosa* as a model organism for T6SS because of its relevance in enhancing our understanding of this field. Moreover, the identification of T6SS elements in *P. aeruginosa* model strains has been key to the identification of T6SS elements in other *Pseudomonas* species. Therefore, further studying T6SSs in *P. aeruginosa* enhances our ability to study and leverage T6SSs in non-pathogenic *Pseudomonas*.

### 4.3. Prevalence of T6SS in Pseudomonas

T6SSs are estimated to be encoded in the genomes of over 25% of Gram-negative bacteria [44]. To test the prevalence of T6SSs in *Pseudomonas*, we used 122 genomes of *Pseudomonas* downloaded from the National Center for Biotechnology Information (NCBI) genome database. The dataset contains representatives of different environments, lineages, and lifestyles, including 45 genomes from a *Pseudomonas* collection extensively cataloged for biocontrol and plant growth-promoting characteristics [58,59,60,61]. We then used McSyfinder’s TXSScan [62] to identify all putative secretion systems encoded in the genomes (Figure S1). We also used the *tssB* sequences for the cluster classification of T6SS loci present in *Pseudomonas* genomes (Figure S2).

**Figure 2:**
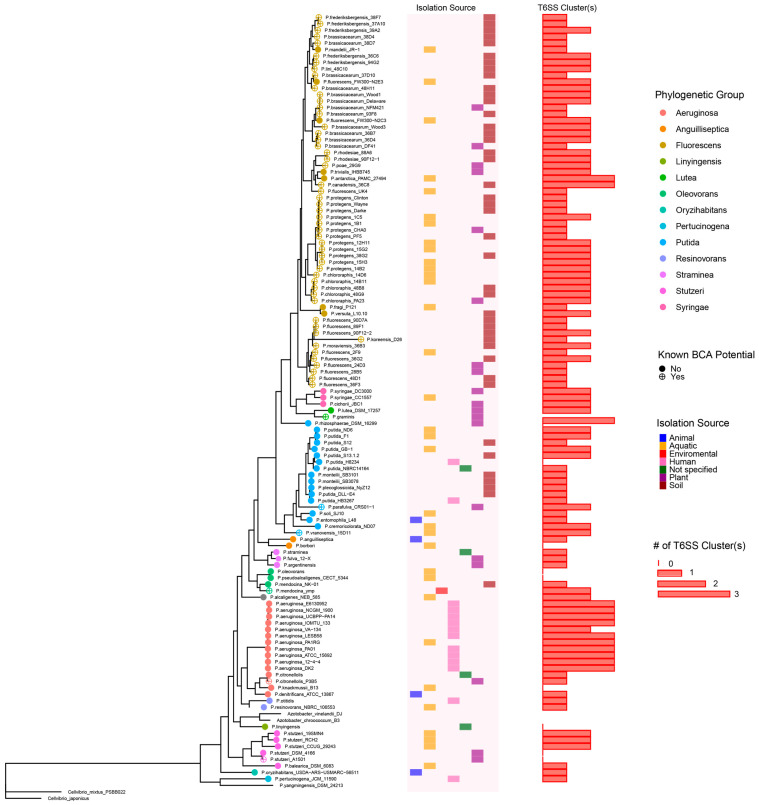
Phylogenetic tree of the *Pseudomonas* spp. Phylogenetic groups, known BCA potential, isolation source, and number of T6SS loci are depicted. Two *Cellvibrio* spp. were used as an outgroup. Two *Azotobacter* spp. were also included in this analysis as these have been shown to fall into the *Pseudomonas* genus. The tree was constructed with concatenated alignments of four housekeeping genes, *16S*, *gyrB*, *rpoB*, and *rpoD*, using the program MrBayes. The number of T6SSs was determined using McSyfinder’s TXXScan. Tree visualization was performed using ggtree 3.4.4.

Here, we show that 111 of the 122 (91%) of the *Pseudomonas* genomes contain at least one T6SS cluster, and 62 of those genomes (51% of the total genomes analyzed) encoded more than one T6SS cluster (Figure 2). Furthermore, cluster classification revealed that these are distributed among five of six known clade groups [45,52], which suggests that these were acquired through horizontal gene transfer (Figure S2). The prevalence of T6SS encoded in *Pseudomonas* genomes is much higher than the estimated 25% proposed by Bingle et al. 2009 [44]. This high prevalence indicates that these T6SSs play a key role in the survival and fitness of *Pseudomonas* in a wide variety of ecosystems. Additionally, the presence of multiple clusters in one genome could potentially lead to a larger variety of T6SS functions within an individual strain of *Pseudomonas*.

## 5. T6SS Studies in Biocontrol *Pseudomonas*

In recent years, studies exploring the role of the T6SS of *Pseudomonas* with biocontrol applications have unveiled intriguing ways in which the T6SS confers a competitive edge against phytopathogens and other microbial competitors. In this section, we delve into a selection of key studies that have enhanced our understanding of how T6SS operates in known biocontrol *Pseudomonas* (see Table 1).

### 5.1. Pseudomonas putida

*Pseudomonas putida* strains are frequently isolated from soils, plants, and polluted sites. Representatives of the species have been implicated in bioremediation, plant growth promotion, the biocontrol of phytopathogens, and other biotechnological purposes [63]. *P. putida* strains have been shown to inhibit phytopathogenic bacteria [64], fungi [65,66], and nematodes [67] both in vitro and in planta. In addition to their wide metabolic capabilities, studies have shown that strains of *P. putida* also harbor T6SS clusters [49,68]. A recent study by Bernal et al. [68] identified three T6SS gene clusters (termed K1-, K2-, and K3-T6SS) and putative effector/immunity proteins in *P. putida* KT2440, a model *P. putida* strain. Furthermore, the study demonstrated that *P. putida* KT2440 can inhibit a broad range of bacteria and reduce necrosis caused by *Xanthomonas campestris* in *Nicotiana benthamiana* leaves in a K1-T6SS-dependent manner, suggesting a role of K1-T6SS as a potent antibacterial weapon.

Subsequent studies have also used *P. putida* strains to elucidate regulatory factors involved in T6SS functioning. A set of studies by Wang et al. [69], Nie et al. [70], and Bernal et al. [71] show a link in the expression of several transcriptional regulators and T6SS in *P. putida*. Wang et al. [70] utilized mutant Δ*fleQ* and Δ*rpoN*, both of which are transcription regulators, the former being involved in flagella and biofilm synthesis [72] and the latter being a sigma factor involved in nitrogen assimilation, stress response, and other cellular functions [73,74]. Downregulation of *fleQ* led to an increase in *hcp1* expression as well as genes involved in c-di-GMP degradation. Similar observations were made by Nie et al. [70], who observed that the c-di-GMP-producing Wsp signal transduction system oppositely regulated T6SS-antibacterial activity and increased biofilm formation via FleQ-FleN in *P. putida*. Bernal et al. [71] used a wide range of mutant *P. putida* KT2440 variants with downregulated expression of several transcription regulators (GacS, RetS, RpoS, RpoN, TurA, and FleQ) to elucidate their role in K1-T6SS expression. Interestingly, the findings of the study showed that K1-T6SS expression is repressed by FleQ, RpoN, and RetS and is positively regulated by GacS. The study also demonstrated a higher expression of K1-T6SS in the stationary phase of growth compared with the exponential phase. Combined, these studies help us better understand the regulatory complexity of T6SSs and suggest that T6SSs are likely to be used during specific circumstances; moreover, in the case of the *P. putida* strains used in these studies, the findings suggest that biofilm formation and the exponential growth phase compete with T6SS functioning.

### 5.2. Pseudomonas protegens

*Pseudomonas protegens* strains are another group of *Pseudomonas* that have commonly been associated with plant ecosystems and biocontrol activity. Model strains such as CHA0 and Pf-5 have been used in a myriad of studies to demonstrate the biocontrol potential of *Pseudomonas* in these species group [7,75,76]. These strains have also been shown to harbor T6SS clusters, and their use in T6SS studies has been valuable for effector discovery and for elucidating T6SS functioning. Whitney et al. [42] first identified the T6SS-employed peptidoglycan glycoside hydrolase effector/immunity families in *P. protegens* Pf-5 using in silico and proteomic approaches. Another family of T6SS effectors, the NADase family, was described in Tang et al. [43], which identified Tne2, a NAD(P)^+^-hydrolyzing effector. Both studies also demonstrated the antibacterial nature of these effectors using competition assays. The latter study by Tang et al. [43] also showed that RhsA (a T6SS DNAse effector) played a role in interspecies competition against *P. putida* KT2440. 

An interesting trait found in *P. protegens* strains is their ability to produce compounds with insecticidal activity, a biocontrol feature that seems to generally be present in *P. protegens* and *P. chlororaphis* strains [77,78]. A recent study by Vacheron et al. [79] demonstrated that T6SS in *P. protegens* CHA0 plays a role in pathogenesis against larval *Pieris brassicae*. In particular, the study showed that *P. protegens* CHA0 invasion of *P. brassicae* gut changed the gut microbiome, which mainly occurring by displacing bacterial members of the Enterobacteriaceae family, suggesting that T6SS is involved in facilitating colonization and pathogenesis by displacement of competitive bacteria.

### 5.3. Pseudomonas fluorescens

*Pseudomonas fluorescens* are yet another group of *Pseudomonas* known for their plant growth-promoting and pathogen suppression capabilities. Substantial contributions to the understanding of T6SS in *P. fluorescens* have come via studies conducted by members of Dr. Annabelle Merieau’s laboratories [80,81,82,83]. Decoin et al. [80] used *P. fluorescens* MFE01, which secreted an abundance of Hcp and VgrG proteins when grown in liquid media. The study utilized mutant Hcp2 knockouts to confirm the antibacterial nature of hcp2 protein secreted by *P. fluorescens* MFE03 against phytopathogenic *Pectobacterium atrosepticum* using both contact-based competition assays in vitro and co-inoculation of potato tubers in planta. The results obtained in the study validate that hcp2 protein confers antibacterial activity against *P. atrosepticum* both in vitro and in planta. Furthermore, the study proved the ability of immunity proteins to confer resistance to T6SS effectors by introducing T6SS immunity proteins present in *Serratia marcescens* into previously susceptible *E. coli* cells.

A subsequent study by Decoin et al. [81] further expanded the relevance of Hcp proteins in *P. fluorescens* MFE01. Mutation of Hcp1 led to reduced mucoidy and loss of flagella, establishing a link between T6SS and motility in *P. fluorescens* MFE01. Additionally, it also showed that Hcp1 contributed to reduced motility of *P. fluorescens* MFN1032, suggesting that T6SSs also mediate intraspecies competition. Gallique et al. [82] identified the presence of another Hcp protein (Hcp3) in *P. fluorescens* MFE01. Hcp3 was then shown to play a role in reducing *P. fluorescens* strains MFN1032 and MFP05 populations when co-cultured with MFE01. Moreover, all three Hcp proteins (Hcp1-3) were implicated in biofilm maturation. Bouteiller et al. [83] built off results observed by Decoin et al. [81] and Gallique et al. [82] by using transcriptomic analysis and electron microscopy to unravel the relationship between flagellar genes and T6SS. The study revealed the absence of flagellar filaments and reduced transcription of flagellar genes in MFE01∆*hcp1* and MFE01∆*tssC* mutants. The combined results obtained from these experiments highlight the complexity of T6SSs and their potential involvement in processes involving cell aggregation (biofilm), motility, and inter- and intra-species bacterial competition. 

### 5.4. Pseudomonas chlororaphis

*Pseudomonas chlororaphis* are common inhabitants of the soil and plant tissue and have been extensively reported for their phytostimulant and biocontrol capacities. *P. chlororaphis* PCL1606, a model strain for biocontrol studies [84], was used in two studies to study T6SS-mediated interactions between PCL1606 and another plant-beneficial bacterium in *Bacillus subtillis* [85,86]. The first study showed a T6SS-mediated predation of *B. subtillis* cells that lack an extracellular matrix. Conversely, the extracellular matrix of *B. subtillis* conferred resistance to this predation, revealing alternate defense mechanisms against T6SEs than the use of cognate immunity proteins. Furthermore, the study showed that this T6SS-led predation of *B. subtillis* by PCL1606 led to sporulation in *B. subtillis*, potentially serving as a survival mechanism. The subsequent study by Perez-Lorente et al. [86] identified Tse1, a hydrolase, and the T6SE involved in degrading the peptidoglycan wall and inducing sporulation in *B. subtillis*.

**Table 1:** Studies involving T6SS in non-pathogenic *Pseudomonas*.

Species and Type Strain	Findings	References
*Pseudomonas protegens* Pf-5	Reported the secretion of an amidase effector (Tae3) in a T6SS-dependent manner	[40]
*Pseudomonas protegens* Pf-5	Identification of T6SS peptidoglycan hydrolase family of effectors and immunity proteins	[42]
*Pseudomonas protegens* Pf-5	Identification of two T6SS effectors: Tne2 (NADse family of effectors) and Rhs2 (DNase family of effectors)	[43]
*Pseudomonas protegens* CHA0	T6SS contributes to colonization in the gut of *Pieris brassicae* by disrupting populations of commensal bacteria	[79]
*Pseudomonas putida* KT2442	FleQ and RpoN negatively regulate T6SS	[69]
*Pseudomonas putida* KT2440	T6SS-mediated inhibition of phytopathogenic *Xanthomonas campestris* in *Nicotiana benthamiana* leaves. Reported secretion of Tke2, a toxic Rhs-type effector, secreted in a T6SS-dependent manner	[68]
*Pseudomonas putida* KT2440	Discovered a class of structural components that associate with TssA for sheath stabilization	[22]
*Pseduomonas putida* KT2440	Increased levels of c-di-GMP produced by the Wsp system decreased T6SS antibacterial activity and increased FleQ-FleN-dependent biofilm formation	[70]
*Pseduomonas putida* KT2440	Regulation of T6SS is linked to global transcription regulators GacS, RetS, RpoS, RpoN, TurA, and FleQ	[71]
*Pseudomonas fluorescens* Pf29Arp	Reported differential expression of T6SS genes in healthy and necrotic wheat roots	[87]
*Pseudomonas fluorescens* MFE01	Evidenced the relationship between Hcp secretion (*hcp2*) and inhibition of the phytopathogen *Pectobacterium atrosepticum*	[80]
*Pseudomonas fluorescens* MFE01	Demonstrated loss of function in the *hcp1* mutant led to decreased mucoidity and loss of flagella	[81,83]
*Pseudomonas fluorescens* MFE01	Demonstrated Hcp proteins in *P. fluorescens* are involved in antibacterial activity and the disruption of biolfim formation in other *Pseudomonas* strains Reported reduced biofilm formation in a mutant knockout of the *tssC* gene	[82]
*Pseudomonas fluorescens* F113	Rhizosphere colonization was severely impaired in T6SS-defected mutants of *P. fluorescens* due to reduced competitive fitness	[88]
*Pseudomonas chlororaphis* P3	Detected upregulation of T6SS-related genes during the production of phenazine-1-carboxamide	[89]
*Pseudomonas chlororaphis* PLC1606	Demonstrated activation of T6SS in *P. chlororaphis* led to sporulation of *Bacillus subtilis*	[85,86]
*Pseudomonas* sp. JY-Q	Reported dual roles of the T6SS effector TseN, which played a role in bacterial competition and nicotine degradation	[90]

## 6. Translating T6SS in Biocontrol *Pseudomonas*

**Figure 3:**
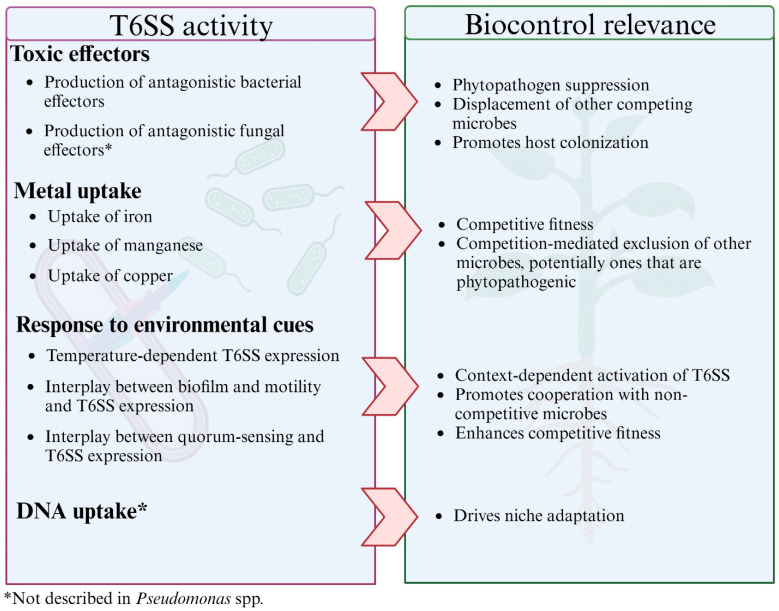
Relevance of T6SS activity in biocontrol applications. Created using BioRender.com (accessed on 10 September 2023).

Studies of type VI secretion have revealed its importance in the overall fitness of *Pseudomonas* spp., and type VI secretion has emerged as a potent mechanism for enhancing *Pseudomonas* spp.’s biocontrol potential (Figure 3). One key feature is the T6SS-mediated inhibition of other phytopathogenic microbes that are predominantly bacterial in nature. With recent evidence elucidating the presence of T6SS antifungal effectors [38], the possibility of T6SEs that can target non-bacterial phytopathogens could expand the scope of T6SS in biocontrol *Pseudomonas* and should be further explored. In addition to pathogen suppression, T6SEs facilitate microbial competition in plant ecosystems, enhancing plant colonization, persistence, and survival of *Pseudomonas* with biocontrol applications [88]. 

Contact-independent uptake of metal ions could also play a role in depriving other microbes of essential nutrients, potentially further suppressing their growth and viability. Furthermore, T6SS has been implicated in the DNA uptake of lysed prey cells facilitating horizontal gene transfer [91]. The phenomenon has not yet been described in *Pseudomonas*, and more studies are needed to elucidate the mechanism behind T6SS-mediated DNA uptake. Nonetheless, it presents the possibility of T6SS facilitating the acquisition of genes that confer niche adaptation and enhance survival.

### 6.1. Future Avenues of T6SS Research

Advancing our understanding of the role of T6SSs in *Pseudomonas* with biocontrol potential presents an interesting avenue of research that can lead to better deployment of these strains as BCAs. Further understanding of effector and immunity protein content in *Pseudomonas* is of particular interest, as these are instrumental to interbacterial competition and thus may play a role in a BCA’s ability to survive in different ecosystems, habitats, and agricultural production systems. Furthermore, understanding the mechanism(s) for suppression that involves T6SSs will improve our understanding of how phytopathogens can be controlled and will help us to understand the long-term durability and/or non-target effects that could result due to the application of the BCAs. Continuous efforts to elucidate T6SEs will also aid in expanding our knowledge of the functions associated with T6SSs. The potential of T6Ses to target eukaryotic organisms such as plants, fungi, oomycetes, nematodes, and insects presents an interesting avenue of research as agricultural production yield reducers are represented by all kingdoms of life. 

Another interesting avenue of study is the interplay between metabolite production and T6SS activity. *Pseudomonas* are known for their metabolic diversity, and in biocontrol *Pseudomonas*, metabolite production has been an emphasis of study due to their capacity to be toxic and be leveraged for phytopathogen suppression. There is evidence that T6SS in *Pseudomonas* may be involved in the secretion of pyoverdine [92], a strong iron chelator, and phenazine-1-carboxamide [89], a metabolite with antibiotic properties. Despite this evidence, it is still unclear what the underlying mechanisms behind these phenomena are, and our understanding of the interplay between metabolite production and T6SS remains limited. Does metabolite production and T6SS activity act synergistically as defensive/offensive mechanisms of microbial competition? Are these oppositely regulated, as seen in some instances occurring between biofilm production and T6SS activity? 

T6SS activity may also be relevant when thinking of community and/or consortia applications of biocontrol *Pseudomonas*. Although most biopesticides consist of a single strain, consortia and community variations have become of interest as there is evidence that these can confer enhanced pathogen suppression and survival [93,94,95]. They also present the opportunity to combine strains that have different modes of action for biocontrol activity, such as creating blends of different toxic metabolites. T6SS-mediated competition has been shown to occur even among closely related strains. Hence, T6SS activity should be considered when creating such consortia, and understanding T6SS dynamics in *Pseudomonas* may be fundamental for their development and subsequent deployment. Furthermore, given the interbacterial activity controlled in part by T6SS found in *Pseudomonas* and other Gram-negative bacteria, we hypothesize that T6SSs may contribute to the mechanism(s) that governs microbiome assemblage and its long-term durability. Application of *Pseudomonas* as biocontrol agents will need to be further researched to determine if having foreknowledge of the T6SSs and their cognate effector/immunity proteins within a *Pseudomonas* biocontrol agent can be used to predict not only conflicts with phytopathogens but also compatibility and long-term durability with the microbiomes of the environment in which it is applied.

### 6.2. Limitations of T6SS Research

Certain limitations should be considered in the study and deployment of T6SSs. Although we demonstrate the high prevalence of T6SS in *Pseudomonas* genomes, many of these are yet to be functionally characterized. Functional characterization may prove to be difficult as the expression of T6SS may be context-dependent and may only be activated or utilized when specific unknown conditions (i.e., favorable environment, presence of competitors, or microbiome assemblage) are met. Furthermore, potential discrepancies between T6SS in vitro activity and activity observed in relevant ecosystems may differ, emphasizing the importance of understanding the underlying mechanisms for T6SS activation and utilization. Ongoing efforts to elucidate the regulation, expression, and activation of T6SSs will aid in addressing these issues, potentially providing better test parameters for the functional characterization of T6SSs.

## 7. Conclusions

The studies reviewed here demonstrate the ability of *Pseudomonas* to utilize T6SS to inhibit bacterial phytopathogens and improve the colonization of their host. In addition, T6SS activity in *Pseudomonas* may be tightly regulated in conjunction with other important survival mechanisms such as biofilm and motility, suggesting a coordinated approach towards survival and or thriving under different circumstances. The high prevalence of this secretion system in *Pseudomonas*, regardless of lifestyle and species designation, suggests that these are important to the fitness and survival of *Pseudomonas* in various environments. The benefits of possessing T6SS are evident as it acts as a dynamic weapon to fend off competing microbes, which allows for the translocation of toxic effectors into target cells. It can also potentially enhance fitness through other non-toxic functions such as the uptake of metal ions and DNA material, though the latter is yet to be reported in the *Pseudomonas* genus. 

Hence, it seems that T6SSs in *Pseudomonas* play a pivotal role in biocontrol success. While *Pseudomonas’s* biocontrol prowess has traditionally been attributed to the production of antagonistic metabolites and their capacity to stifle pathogen growth by directly inhibiting or outcompeting phytopathogens for resources and space, it is imperative to delve into the potential of T6SSs for enhancing *Pseudomonas’s* biocontrol capabilities. A deeper comprehension of T6SS interactions within these *Pseudomonas* strains holds the promise of refining their utility as biological control agents (BCAs). This could be achieved through heightened efficacy in phytopathogen control and by potentially bolstering their resilience in cropping systems by pinpointing specific effector/immunity proteins that are antagonistic toward pathogens while simultaneously identifying those that align more harmoniously with the existing microbiomes associated with cropping systems. Lastly, we note that although this review primarily delves into various aspects of *Pseudomonas* T6SSs and places emphasis on the intersection between T6SS and biocontrol applications in this genus, it is also important to note that the versatility of this secretion system extends well beyond this field. Numerous reviews have comprehensively covered these various aspects, containing information from T6SS functioning and regulation to its relevance in various ecological niches [96,97,98,99,100,101,102,103].

## Data Availability

Not applicable.

## Supplementary Materials

The following supporting information can be downloaded at: https://www.mdpi.com/article/10.3390/microorganisms11112718/s1, Figure S1: Secretion systems and related appendages in *Pseudomonas* spp.; Figure S2: *TssB* structural gene tree based on translated amino acid sequences.
