# Supplementary material for: T6SS: A Key to Pseudomonas’s Success in Biocontrol?
Source: Microorganisms. 2023 Nov 7;11(11):2718. doi: 10.3390/microorganisms11112718 (PMC10673566; doi:10.3390/microorganisms11112718)

Figure S1: Secretion systems and related appendages in *Pseudomonas* spp. T6SS core components are in red. McSyfinder's TXXScan was used for the identification of these components. ComM – Competence development, Flg – Flagellin, MSH - Mannose-sensitive hemagglutinin, T1SS - Type I, T2SS - Type II, T3SS - Type III, T4 Pili - Type IV Pili, T4SS - Type IV, T5SS - Type V, **T6SS - Type VI**, Tad – Tight adherence.

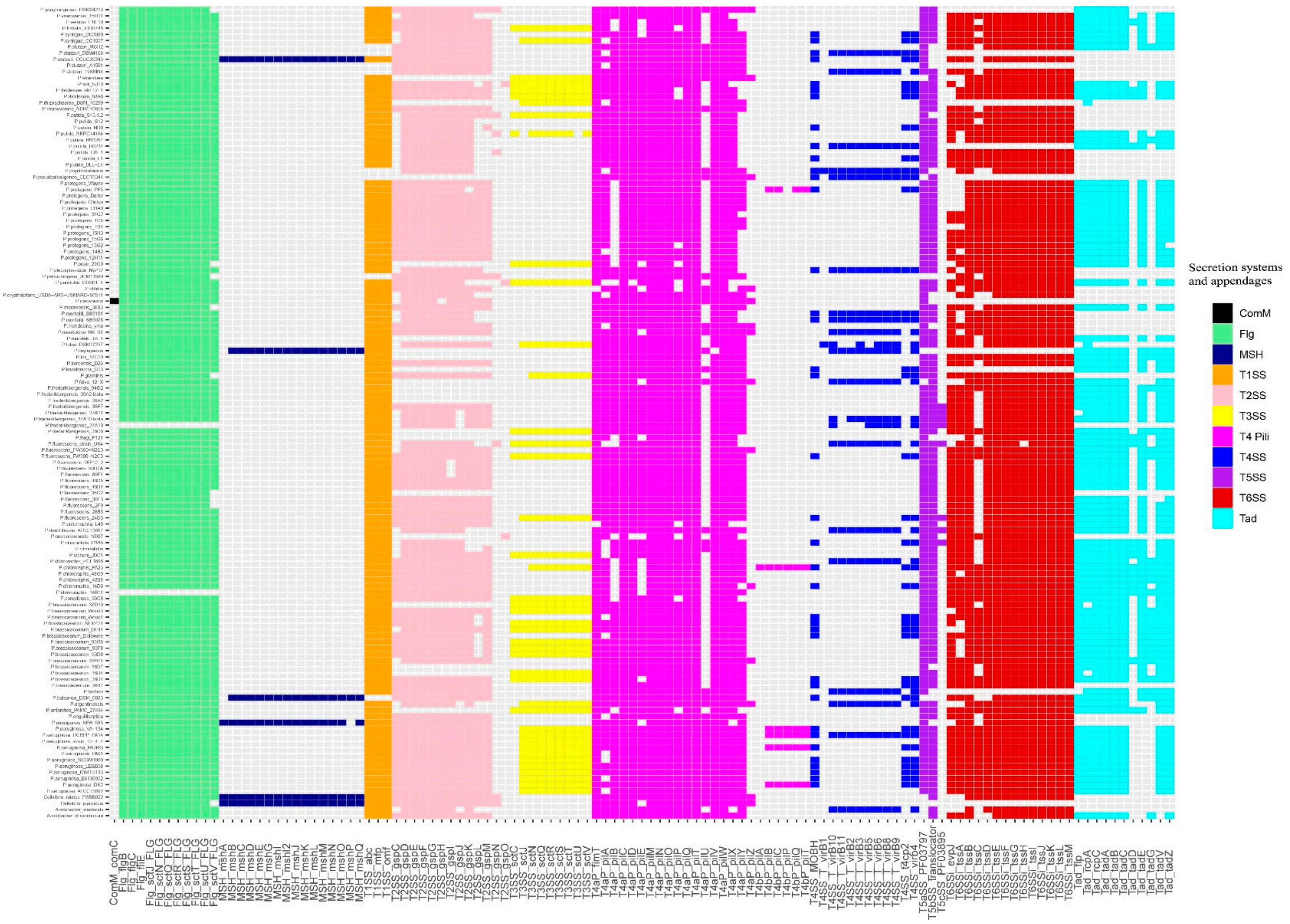

Figure S2: *TssB* structural gene tree based on translated amino acid sequence. Clades I-V are depicted. Tree was generated using concatenated alignments of *tssB* sequences extracted from T6SS loci encoded in *Pseudomonas* genomes using FastTree 2.1.

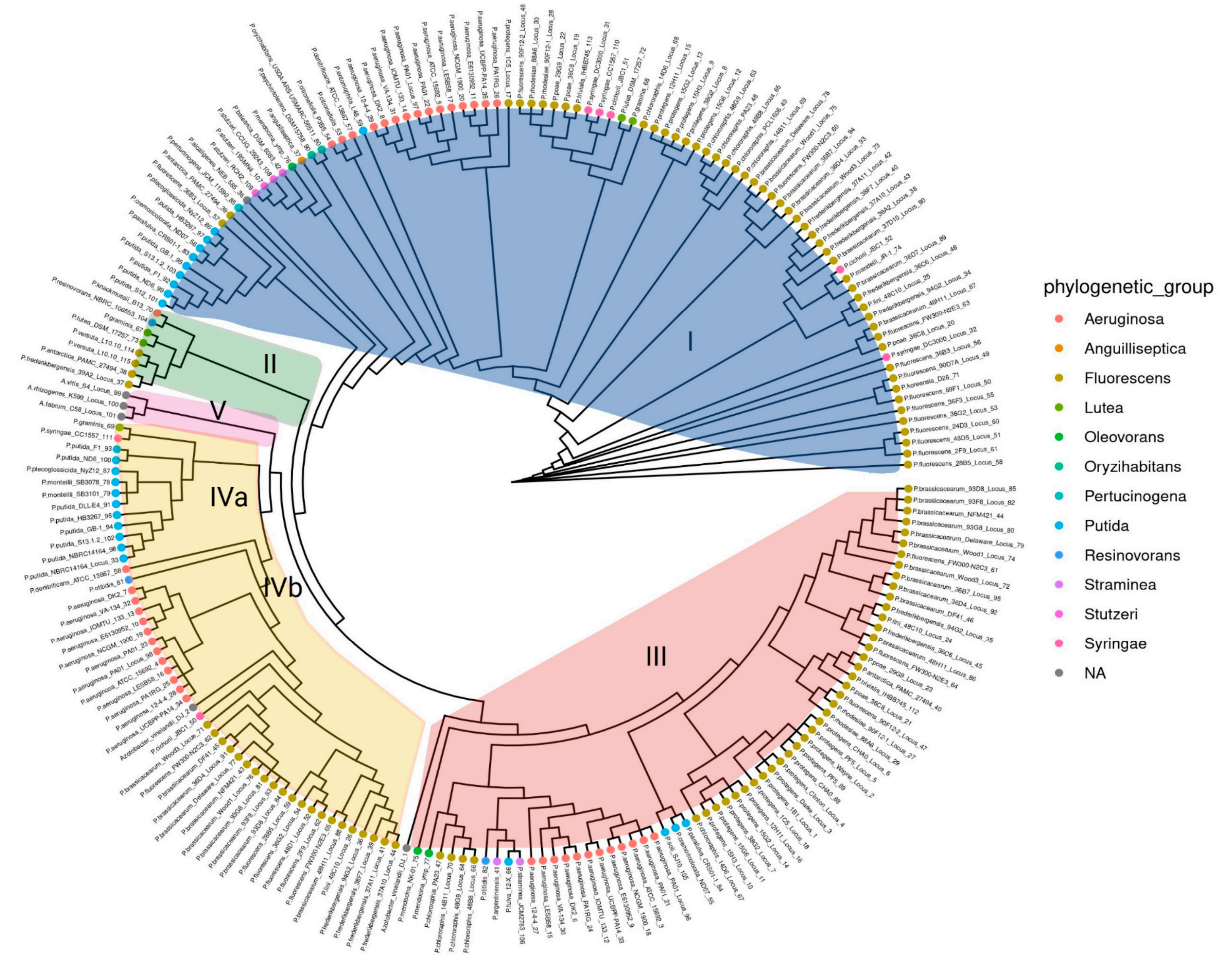

Supplement: Supplementary file 1 [file microorganisms-11-02718-s001.zip › microorganisms-2656015-supplementary.pdf]
